# Microbiome Analysis of the Bamboo Aphid *Melanaphis bambusae* Infected with the Aphid Obligate Pathogen *Conidiobolus obscurus* (Entomophthoromycotina)

**DOI:** 10.3390/insects13111040

**Published:** 2022-11-10

**Authors:** Tian Yang, Xiaojun Wang, Xiang Zhou

**Affiliations:** State Key Laboratory of Subtropical Silviculture, School of Forestry and Biotechnology, Zhejiang A&F University, Hangzhou 311300, China

**Keywords:** aphid pest, QIIME, microbiome, uncultured method, Entomophthorales

## Abstract

**Simple Summary:**

Bamboo are widespread fast-growing perennials with multiple economic and ecological values. Bamboo aphids are the main pests that threaten bamboo forests. The naturally occurring entomopathogenic fungi in these forests are locally effective in controlling aphid populations, especially during monsoon. This study investigated the community structures of bacteria and fungi in the bamboo aphid *Melanaphis bambusae* and their changes under the stress of fungal infection or starvation. Results showed a difference in community structure between the infected and starved aphids. Moreover, the relative abundance of several operational taxonomic units increased dramatically in response to infection by *Conidobolus obscurus*. This implied that the aphid-borne microbes possibly facilitated the infection process, highlighting its potential for improving the control efficacy of fungal biological agents on bamboo aphids.

**Abstract:**

Insect-associated microbes exert diverse effects on host fitness. This study provides insights into the microbiota of the bamboo aphid, *Melanaphis bambusae,* and their response to *Conidiobolus obscurus* infection. 16S rRNA and ITS sequencing data were used to analyze the bacterial and fungal samples associated with healthy, infected, and starved aphids. At ≥97% nucleotide similarity, the total reads were clustered into 79 bacteria and 97 fungi operational Taxonomic Units (OTUs). The phyla Proteobacteria and Ascomycota dominated the bacterial and fungal communities, respectively. The significant divergence in OTU distribution presented differential profiles of the microbiota in response to host conditions. Lower α-diversity indices were found in bacterial and fungal diversity when the aphids were experiencing fungal infection and starvation stresses, respectively. The β-diversity analyses of the communities showed significant differences among the three host conditions, demonstrating that aphid-associated microbiota could significantly shift in response to varying host conditions. Moreover, some OTUs increased under fungal infection, which potentially increased aphid susceptibility. Presumably, *C. obscurus* infection contributed to this increase by causing the disintegration of host tissues other than host starvation. In conclusion, understanding the differentiation of aphid microbiota caused by fungal entomopathogens helped facilitate the development of novel pest management strategies.

## 1. Introduction

Insect-associated microbiota have been studied for their benefits in host adaptation strategies such as nutrition acquirement, detoxification, and resistance towards natural enemies or pathogens [1,2,3,4,5,6,7,8]. Some studies have proposed the use of endosymbionts, such as cytoplasmic incompatibility-inducing *Wolbachia*, as biological control agents for insect pests [9,10]. Undoubtedly, the diversity of insects and their symbionts contribute to the complexity of insect–host-microbe interactions [11]. A detailed study of insect-inhabiting microbes and variation in their community structure in response to environmental stresses and host conditions is a novel approach to unveil their obscure relationships [12].

Aphids are economically important pests in agroforestry. Most studies on the symbionts of aphids are related to the species *Acyrthosiphon pisum* and its bacterial symbionts, showing that the community composition includes the obligate symbiont *Buchnera aphidicola* and eight heritable facultative symbionts [4,6,13]. Considering the comparatively weakened immune system of aphids, symbionts exert significant effects to protect the hosts against natural enemies [14]. *Regiella insecticola* has been reported to increase host resistance towards the aphid-obligate pathogen *Pandora neoaphidis* (Entomophthoromycotina) and lower the transmission rate of this fungal pathogen in aphid populations [15]. *Serratia symbiotica* and *Hamiltonella defensa* have various effects on host resistance to parasitoids [16,17]. Fungi within aphids have been reported in a few species, such as yeast-like symbionts [18,19], but the underlying interactions between them have rarely been studied.

Fungal entomopathogens in Entomophthoromycotina (Zoopagomycota) infect arthropod hosts via conidia germinating on the cuticle and the germ tube penetrating into the haemocoel, multiplying, and rapidly exhausting the host’s nutritive materials [20,21]. Successful colonization of the host through physical and biological barriers is a prerequisite for infection [22]. Fungi produce diverse hydrolytic enzymes, such as subtilisin-like serine proteases, to breach host tissues [23,24,25,26], but the influence of such pathogens’ infection on the microbiota of the host has rarely been studied. The host tissue disintegration and nutrient exhaustion caused by fungal proliferation dramatically change the host’s internal environment, possibly resulting in significant variation among the community composition of the host’s microbes.

To determine the microbiome response to mycosis, this study investigated the bacterial and fungal communities in the economically important bamboo aphid *Melanaphis bambusae* (Fullaway) under infection stress imposed by the aphid-obligate pathogen *Conidiobolus obscurus* from Entomophthoromycotina. Overall, this study provides preliminary insights into the subtle relationships between aphid hosts, their microbiota, and obligate aphid pathogens.

## 2. Materials and Methods

### 2.1. Fungal Isolation and Culture

*C. obscurus* isolate ARSEF 7217 was acquired from the USDA-ARS Collection of Entomopathogenic Fungal Cultures (Ithaca, NY, USA) and stored at −80 °C for long-term cryopreservation [27]. The isolate was recovered by host passage through the bamboo aphid *M. bambusae* before conidial inoculation. *C. obscurus* was cultured on the enriched medium of Sabouraud dextrose agar supplemented with yeast extract (glucose 40, peptone 10, yeast extract 10, and agar 15 g L^−1^) for 4 days, incubated at 24 °C, and 12:12 h light: dark (L:D) photoperiod. To obtain homogenized mycelial material, the culture pieces from the solid medium were transferred into 50 mL liquid medium of Sabouraud dextrose broth with yeast extract, incubated in a 150 mL flask, and shaken at 120 rev min^−1^ for 4 days at 24 °C. The mycelium was filtered and tiled evenly on 90 mm diameter Petri dishes. After removing residual water with sterile paper, the mycelium-inclusive plates were maintained for 12 h at 24 °C for sporulation and were deemed ready for inoculating the aphids using a conidial shower [28].

### 2.2. Aphid Rearing and Inoculation

The alate adults of *M. bambusae* were air-captured from the roof of a building in the campus of Zhejiang A&F University using a yellow-cloth-plus-plant trap and reared on potted plants of *Chimonobambusa quadrangularis* (Fenzi) Makino in an open field [29].

The *M. bambusae* adults were transferred to 90 mm diameter leaf-inclusive dishes for *C. obscurus* inoculation, with each dish containing 50 adults. Leaf-inclusive dishes were prepared by embedding fresh detached bamboo leaves on agar (15 g L^−1^) with the foliar undersides exposed for aphid feeding. The sporulating plates were inverted on the aphid-feeding dishes for inoculation, and the inoculation concentration of conidia was controlled at approximately 1000 conidia per mm^2^. A small glass coverslip was placed near the cohort under each exposure condition to assess the number of deposited conidia per mm^2^. To inoculate aphids evenly, the sporulating plates were turned 90° every quarter of inoculation time. Conidia-inoculated aphids were reared at 24 °C and a 12:12 h light: dark (L:D) photoperiod for sampling.

### 2.3. Sampling Healthy, Mycotized, and Starved Aphids

Live aphids were prepared by collecting healthy adult aphids feeding on potted *C. quadrangularis* plants in three sterile Eppendorf tubes (100 mg per tube) as replicates and frozen in liquid nitrogen for bacterial and fungal genomic DNA extraction. Fresh mycotized aphid cadavers were collected 48 h post-inoculation in three sterilized Eppendorf tubes (100 mg per tube), frozen in liquid nitrogen, and stored at −80 °C. Another batch of healthy adults collected in three sterilized Eppendorf tubes (100 mg per tube) was maintained under starvation stress with other conditions being ambient for 48 h and then frozen in liquid nitrogen.

### 2.4. DNA Extraction and Sequencing

The collected aphids were used for DNA extraction, as the microbes on the host cuticles interacted with *C. obscurus*. DNA samples were extracted from each tube of 100 mg aphids or cadavers using the MiniBEST Universal Genomic DNA Extraction Kit (Ver. 5.0, TakaRa, Tokyo, Japan) according to the manufacturer’s instructions. The sampled DNA concentrations were measured using a NanoDrop2000 (Thermo fisher Scientific, New York, NY, USA). DNA pools from healthy aphid samples were analyzed for bacterial (HAB) and fungal (HAF) communities. The bacteria (MAB and SAB) and fungi (MAF and SAF) from mycotized aphid cadavers and starved aphids were also analyzed. Total DNA pools were then frozen on dry ice and transported to Novogene Co. (Beijing, China) for sequencing. We sequenced either the V4 region of 16S rRNA gene or the internal transcribed spacer-1 (ITS1) region of rDNA using the Illumina MiSeq platform to analyze the bacterial or fungal community composition under the conditions of healthy, mycotized, and starved aphids.

### 2.5. Sequence Clustering and Annotation

The sequences obtained from the MiSeq runs were demultiplexed based on the barcode sequences using the default script in Quantitative Insights into Microbial Ecology (QIIME, version 1.8.0). Chimeric sequences were detected using usearch61 and filtered. The remaining sequences were clustered into operational taxonomic units (OTUs) based on ≥97% pairwise identity using the tIOTU picking strategy in QIIME. The representative sequence for the Ich OTU was aligned using PyNAST in QIIME, and taxonomic classification of the representative sequence was conducted using the default uclust consensus taxonomy assigner in QIIME against the Greengenes database (version 13_8) and by using the megablast algorithm against the NCBI nucleotide collection (nr/nt). Based on OTU abundance and annotation information, the relative abundance of the bacterial and fungal taxa at different taxonomic levels (phylum, class, order, family, and genus) were calculated.

### 2.6. Microbiome Statistical Analyses

For microbiome analysis, the α-diversity of each sample was measured as the estimated species richness calculated using the Chao estimator (1), Shannon (2), and Simpson (3) diversity indices. β-diversity analyses were also conducted to compare differences among the samples using the non-metric multidimensional scaling (NMDS) method based on Bray–Curtis dissimilarities. Heatmaps were generated in line with OTU distribution and abundance classification using the function “plotMRheatmap” from the R package “metagenomeSeq” (https://mirrors.sjtug.sjtu.edu.cn/cran/ (accessed on 31 October 2022). Good’s estimate of coverage was also used to determine the quality of the sequencing results.

(1) $$\mathrm{Chao}=S_{obs}+\frac{n_{1}\left(n_{1}-1\right)}{2\left(n_{2}+1\right)}$$

In the Chao index, *S_obs_* was the observed number of species (OTUs), *n*_1_ was the number of OTUs with only one sequence (i.e., “singletons”), and *n*_2_ was the number of OTUs with only two sequences (i.e., “doubletons”).

(2) $$\;\mathrm{Shannon}\;\mathrm{Diversity}\;\mathrm{Index}=-\sum_{i=1}^{s}\left(p_{i}\ln p_{i}\right)$$

In the Shannon index, *p* was the proportion of read counts of a sequence representing a bacterial or fungal genus (*i*-th) divided by the total read counts of all bacterial or fungal genera in a sample.

(3) $$\;\mathrm{Simpson}\;\mathrm{Index}\;=1-\left[\sum^{\;}n_{i}\left(n_{i}-1\right)/N\left(N-1\right)\right]$$

In the Simpson index, *n_i_* was the proportion of read counts of a sequence representing a bacterial or fungal genus (*i*-th) and *N* was the total read counts of all bacterial or fungal genera in a sample.

## 3. Results

### 3.1. Sequence and Microbiome Statistical Analyses

**Figure 1:**
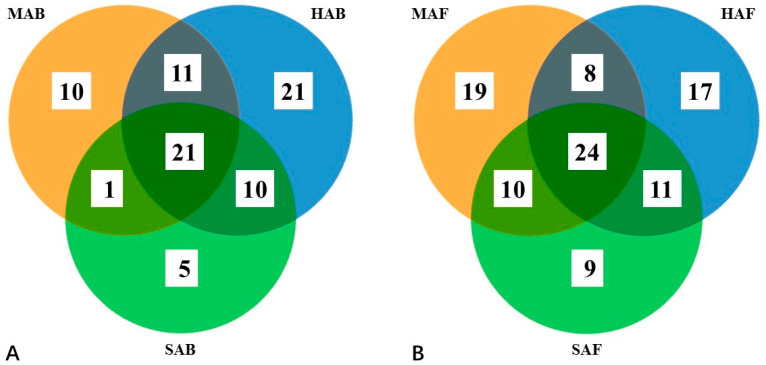
The unique and overlapping bacterial (**A**) and fungal (**B**) OTUs assembled from the three treatment conditions. HAB and HAF indicate healthy aphids, MAB and MAF mycotized aphids, and SAB and SAF starved aphids.

After the removal of aphid- and *C. obscurus*-derived reads, the total number of reads obtained from the DNA pools were 257,915. A total of 100,659 and 157,256 reads were assigned to bacteria and fungi, respectively (Table 1). The sequences were clustered into 176 OTUs with a maximum dissimilarity of 3% in sequence identity; 79 were assigned to bacteria and 97 to fungi. Of these, 26.6% of bacterial and 24.7% of fungal OTUs were present in all three communities under the host conditions of healthy, mycotized, and starved aphids (Figure 1). The rarefaction curves for each DNA pool were almost asymptotic (Figure S1), consistent with the coverage estimates. These results demonstrated that rare taxa were missed by the deep sequencing method. The Chao richness estimators calculated using a random set of sequences in the six different microbial communities indicated their theoretical species richness. The number of OTUs were similar to the Chao estimators for each community, also demonstrating few missed detections of rare species in this study. Both Shannon and Simpson diversity indices, which incorporated evenness and species richness, indicated that the taxonomic diversity of the bacterial community was the highest and lowest under the starvation and infection conditions, respectively; and that of the fungal community was different, being similar under healthy and infected conditions and the lowest under starvation conditions, as shown in Table 1. β-diversity analyses of the bacterial and fungal communities using the NMDS method demonstrated significant differences among the three host conditions (Figure S2).

### 3.2. Taxonomic Distribution of Aphid-Associated Bacterial and Fungal Communities

OTUs were further assigned to different taxa, and their relative abundances were estimated under the three host conditions. HAB and HAF indicated healthy aphids, MAB and MAF mycotized aphids, and SAB and SAF starved aphids. Seventeen known bacterial classes belonging to nine known phyla were identified. All nine phyla were presented in HAB, and four out of the nine phyla were present in both MAB and SAB (Figure S3A). Proteobacteria were dominant in all three conditions: 89.74% in HAB, 98.10% in MAB, and 75.88% in SAB; Bacteroidetes represented 23.64% in SAB, 4.18% in HAB, and 1.00% in MAB. At the class level, γ-proteobacteria were dominant: 83.01% in HAB, 94.24% in MAB, and 68.93% in SAB; followed by Bacilli in HAB (3.47%), β-proteobacteria in MAB (3.04%), and Flavobacteria in SAB (17.34%) (Figure S4A).

Nine known fungal classes belonging to three known phyla were identified, in addition to some unknown groups. Ascomycota was dominant, with 96.37% in HAF, 92.58% in MAF, and 94.60% in SAF (Figure S3B). At the class level, Saccharomycetes dominated, with 55.96% in HAF and 41.15% in MAF. Dothideomycetes were significantly enriched in SAF (76.28%) compared to 35.14% in HAF and 37.02% in MAF (Figure S4B).

**Figure 2:**
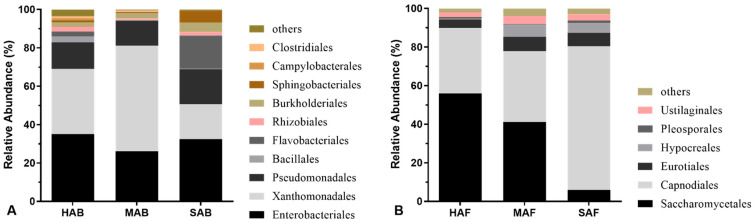
Relative composition of different bacterial (**A**) and fungal (**B**) orders in three aphid conditions. HAB and HAF indicate healthy aphids, MAB and MAF mycotized aphids, and SAB and SAF starved aphids.

At the order level, there were three orders with high abundances in all three bacterial communities: Enterobacteriales, Xanthomonadales, and Pseudomonadales (Figure 2A). Enterobacteriales dominated in HAB (35.06%) and SAB (32.43%), Xanthomonadales were enriched in MAB (55.02%), and Flavobacteriales were abundant (17.34%) in SAB. In the fungal community (Figure 2B), there were differences in the dominant order among the three fungal communities: Saccharomycetales (55.96% in HAF and 41.15% in MAF) and Capnodiales (74.57% in SAF). At the family level, Enterobacteriaceae and Xanthomonadaceae were the dominant bacterial communities, whereas Mycosphaerellaceae and Pichiaceae dominated the fungal communities (Figure S5).

**Figure 3:**
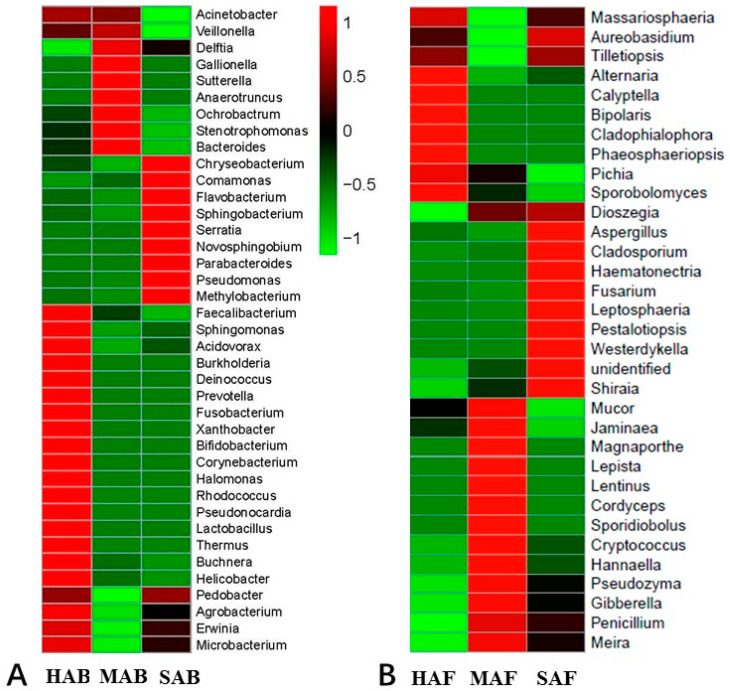
Heat maps showing the bacterial (**A**) and fungal (**B**) genus frequency distribution among the three different conditions of aphids. The different color intensities represent the relative abundance of each genus. HAB and HAF indicate healthy aphids, MAB and MAF mycotized aphids, and SAB and SAF starved aphids.

There was a difference at the genus level in both bacterial and fungal communities. The difference in the relative abundance of the assigned genera presented higher divergence than those presented at higher taxonomic levels or with the change in the microbial community composition in response to different host conditions (Figure 3).

### 3.3. Representative OTUs in Bacterial Communities

Eighteen bacterial OTUs represented ≥1.0% relative abundance among the three communities (Table 2). One OTU related to the genus *Stenotrophomonas* was dominant, and the relative abundance increased in MAB and decreased in SAB compared to that in HAB. The relative abundance of one OTU assigned to the genus *Buchnera* was reduced in MAB and SAB, which is homologous to the well-known aphid endosymbiont, *B. aphidicola*. A similar reduction occurred in four other OTUs, including one enriched OTU related to *Erwinia* sp. Several OTUs were highly abundant in SAB and comparatively low in HAB and MAB, which matched soil- or plant-associated *Flavobacterium* spp., *Acinetobacter* sp., *Sphingobacterium* sp., *Chryseobacterium* sp., and *Pseudomonas* sp. One OTU that was only present in SAB matched *Serratia marcescens* found in the bumble bee gut. Five other OTUs increased in abundance in both MAB and SAB relative to HAB.

### 3.4. Representative OTUs in Fungal Communities

There were 11 fungal OTUs representing at least 1.0% relative abundance (Table 3). One OTU that was highly homologous to *Pichia* sp. was dominant in HAF, reducing MAF and SAF. Three OTUs showed reduced abundance in MAF and increased abundance in SAF, one of which was enriched in SAF and related to *Cladosporium* sp. Four OTUs had a low number of reads in HAF and MAF, but were comparatively high in SAF, which matched different phytopathogenic species. One OTU related to *Fusarium* sp. was highly abundant in MAF and SAF, and another OTU that was enriched only in MAF was homologous to *Penicillium* sp. The other three OTUs increased in relative abundance in MAF and SAF compared to that in HAF, one of which was homologous to the aphid endosymbiont *Pseudozyma aphidis*.

## 4. Discussion

The survival conditions of insect-associated microbes depend on the host for shelter and nutrition supply [30]. The microbial communities shift considerably under the stress of nutritive deficiency caused by either fungal infection or fasting, as presented in this study, which showed significant changes in both bacterial and fungal aphid-associated microbe communities. The numbers of OTUs under starvation conditions were the lowest, implying that the microbial species that were highly dependent on host nutrition did not survive. Fungal infection shifted the community composition, probably due to rapid host tissue breaching and nutritional competition with the symbionts. Considering the low Shannon and Simpson diversity indices in the bacterial community under infection conditions, fungal infection might have influenced the aphid-inhabiting bacterial species more deeply than aphid-inhabiting fungi.

Aphids frequently encounter epiphytic and plant-pathogenic microbes because of their plant-dependent lifestyles, and many such pathogenic species have been identified as members of the aphid gut microbiome [31]. In the present study, many OTUs with relative abundance matched plant-associated microbes, such as *Erwinia* spp., which contain mostly plant-pathogenic species. It has been reported that some phytopathogens that are entomopathogenic, such as *Pantoea stewartii* and *Dickeya dadantii* (*Erwinia chrysanthemi*), could colonize aphid gut and kill the hosts [32,33]. Some virulence factors against host aphids have been reported in these species such as Cyt-like toxin genes in *D. dadanttii.* However, many unidentified virulence factors exist in symbiont microbes [32,34,35]. Interestingly, we found a Cyt-like gene in the *C. obscurus* genome and its relationship with fungal virulence against aphids in a previous study [36]. Based on the analysis of the average G + C percentage of genes, a previous study speculated horizontal gene transfer of the Cyt-like gene between fungal pathogens and other bacterial lineages [25]. The present study might provide support for the horizontal transfer events of virulence-related genes that occur between fungal entomopathogens and bacterial flora in their host aphids. The identification of such virulence factors vectored by microbiota might add to insecticidal gene resources.

Infection and starvation probably weaken the host immune system, favoring an abundance of opportunistic or potential pathogens and facultative saprophytes. Species with increasing abundance under infected conditions might be related to the host’s susceptibility to the fungal entomopathogen [37]. Our results show that the bacterial OTU of *Stenotrophomonas* exceeded 50% in the MAB. It is possible that the *Stenotrophomonas* sp. found in *M. bambusae* took advantage of *C. obscurus* infection. However, the *Stenotrophomonas* OTU decreased in starved aphids, implying that competition from facultative saprophytes inhibited it at the initial phase of host death. Similar phenomena were reported in cadavers of *Photorhabdus*-killed *Galleria mellonella*, which presented a low abundance of *Stenotrophomonas* species in fresh cadavers and was dominant 12 days after host death [38]. We speculated that infection-induced host tissue disintegration, similar to the late phase of host death, favored the proliferation of *Stenotrophomonas* strains. Aggregative behavior and virulence of *Stenotrophomonas* sp. were also observed in a nematode model [39,40]. However, the relationships dynamics between *Stenotrophomonas*, the host aphid, and *C. obscurus* and their potential virulence factors remain obscure.

In conclusion, *C. obscurus* infection distinctly altered the microbiota of bamboo aphids. These results can be used to further the understanding of fungal pathogens invading aphid–host systems and for the future development of novel pest management strategies.

## Figures and Tables

**Table 1:** α-diversity estimation of the 16S rRNA gene and ITS libraries from the Illumina Miseq analysis.

Sample ^a^	Reads	OTUs	Chao	Shannon	Simpson	Coverage
16S rRNA for bacteria						
HAB	40,600	63	63.00	3.28	0.82	1.00
MAB	34,301	42	44.50	2.29	0.66	1.00
SAB	25,758	36	37.50	3.72	0.90	1.00
ITS for fungi						
HAF	79,522	68	64.62	2.11	0.67	1.00
MAF	45,042	61	63.14	2.29	0.64	1.00
SAF	32,692	60	66.12	0.66	0.16	1.00

^a^ HAB and HAF indicate healthy aphids, MAB and MAF mycotized aphids, and SAB and SAF starved aphids.

**Table 2:** Variations of relative abundance (%) of 16S rRNA gene amplicons in aphid-associated bacterial libraries. HAB, MAB, and SAB indicate healthy, mycotized, and starved aphid-associated bacteria, respectively.

Order	Family	Best Match in GenBank	HAB	MAB	SAB
Xanthomonadales	Xanthomonadaceae	*Stenotrophomonas* sp. in nematode (KT151870.1)	34.01	55.02	18.21
Enterobacteriales	Enterobacteriaceae	*Klebsiella* sp. in bioaerosols (KY911397.1)	8.72	15.25	10.23
Pseudomondales	Moraxellaceae	*Acinetobacter* sp. in ant colonies (KT025919.1)	10.99	11.74	10.46
Enterobacteriales	Enterobacteriaceae	*Pantoea* sp., an endophyte (KR094823.1)	0.42	4.53	2.77
Enterobacteriales	Enterobacteriaceae	*Erwinia* sp., an endophyte (KU891828.1)	20.74	4.01	15.8
Enterobacteriales	Enterobacteriaceae	*Buchnera aphidicola*, an endosymbiont of *Aphis craccivora* (EF614236.1)	5.17	2.4	2.15
Burkholderiales	Comamonadaceae	*Delftia lacustris* in fall armyworm gut (KX273063.1)	0.77	1.58	1.24
Pseudomonadales	Moraxellaceae	*Acinetobacter* sp., an endophytic bacterium (KU725922.1)	2.72	1.24	5.56
Burkholderiales	Comamonadaceae	*Comamonas koreensis* in *Monochamus alternatus* gut (KX461916.1)	0.69	0.94	2.85
Sphingobacteriales	Sphingobacteriaceae	*Sphingobacterium multivorum*, an epiphyte (KJ638992.1)	1.22	0.61	5.95
Bacillales	Planococcaceae	*Staphylococcus xylosus* on cotton root (LT797529.1)	3.06	0.14	0.21
Campylobacterales	Helicobacteraceae	*Helicobacter pylri* (AP017348.1)	1.03	0.07	0
Pseudomonadales	Pseudomonadaceae	*Pseudomonas* sp. on tomato root (KY231156.1)	0.11	0.05	2.27
Flavobacteriales	Weeksellaceae	*Chryseobacterium* sp. on guarana root (KT699830.1)	0.89	0.05	3.44
Rhizobiales	Rhizobiaceae	*Agrobacterium tumefaciens* in fire ant (KY874047.1)	1.88	0.05	0.99
Flavobacteriales	Flavobacteriaceae	*Flavobacterium johnsoniae* in soil (HM224403.1)	1.13	0.02	7
Flavobacteriales	Flavobacteriaceae	*Flavobacterium* sp. in soil (KU877342.1)	0.3	0.01	6.86
Enterobacteriales	Enterobacteriaceae	*Serratia marcescens* in bumble bee gut (LT631777.1)	0	0	1.48

**Table 3:** Variations in relative abundance (%) of ITS amplicons in aphid-associated fungal libraries. HAF, MAF, and SAF indicate healthy, mycotized, and starved aphid-associated fungi, respectively.

Order	Family	Best Match in GenBank	HAF	MAF	SAF
Saccharomycetales	Pichiaceae	*Pichia guilliermondii* (EF151440.1)	55.96	41.14	5.88
Capnodiales	Mycosphaerellaceae	*Cladosporium* sp. *Deschampsia caespitosa* seed associated (KX839295.1)	2.88	16.82	11.46
Capnodiales	Mycosphaerellaceae	*Cladosporium* sp. Ericaceae root (KU986780.1)	30.3	16.16	56.96
Hypocreales	Nectriaceae	*Fusarium* sp. in Monarda citriodora flower (KU527799.2)	0.27	6.14	1.05
Eurotiales	Trichocomaceae	*Penicillium* sp. endophytic fungus in lotus leaves (KX722229.1)	1.5	4.84	1.67
Ustilaginales	Ustilaginaceae	*Pseudozyma aphidis* (KM268868.1)	2.26	3.94	3.16
Capnodiales	Mycosphaerellaceae	*Cladosporium* sp., potential pathogen inducing black spot of *Dendrobium officinale* (KY114871.1)	0.87	3.57	6.15
Eurotiales	Trichocomaceae	*Penicillium* sp., Persian oak (KX611014.1)	2.56	2.52	3.6
Eurotiales	Trichocomaceae	*Penicillium* sp., fungal endophyte (KT291052.1)	0.13	0.08	1.1
Hypocreales	Nectriaceae	*Gibberella zeae*, phytpathogen (HQ333195.1)	0	0	2.02
Hypocreales	Nectriaceae	*Fusarium* sp., phytopathogen (KU578346.1)	0.04	0	1.32

## Data Availability

Illumina sequence data have been submitted to CNGBdb (https://db.cngb.org/, accessed on 7 October 2022) under the accession number CNP0003555. All data generated or analyzed during this study are included in this published article and supplementary file.

## Supplementary Materials

The following supporting information can be downloaded at: https://www.mdpi.com/article/10.3390/insects13111040/s1, Figure S1: Rarefaction curves of bacterial (A) and fungal (B) species under the three host conditions. Figure S2: NMDS analysis of aphid-associated bacterial (A) and fungal (B) flora under the three different conditions. Figure S3: Relative composition of bacterial (A) and fungal (B) phyla in aphid-associated microbial communities under the three different conditions. Figure S4: Relative composition of bacterial (A) and fungal (B) classes in aphid-related microbiomes under three different conditions. Figure S5: Relative compositions of different bacterial (A) and fungal (B) families under the three aphid conditions.
