# Supplementary material for: Microbiome Analysis of the Bamboo Aphid Melanaphis bambusae Infected with the Aphid Obligate Pathogen Conidiobolus obscurus (Entomophthoromycotina)
Source: Insects. 2022 Nov 10;13(11):1040. doi: 10.3390/insects13111040 (PMC9692958; doi:10.3390/insects13111040)
Supplement: Supplementary file 1 [file insects-13-01040-s001.zip › insects-1984991-supplementary.pdf]

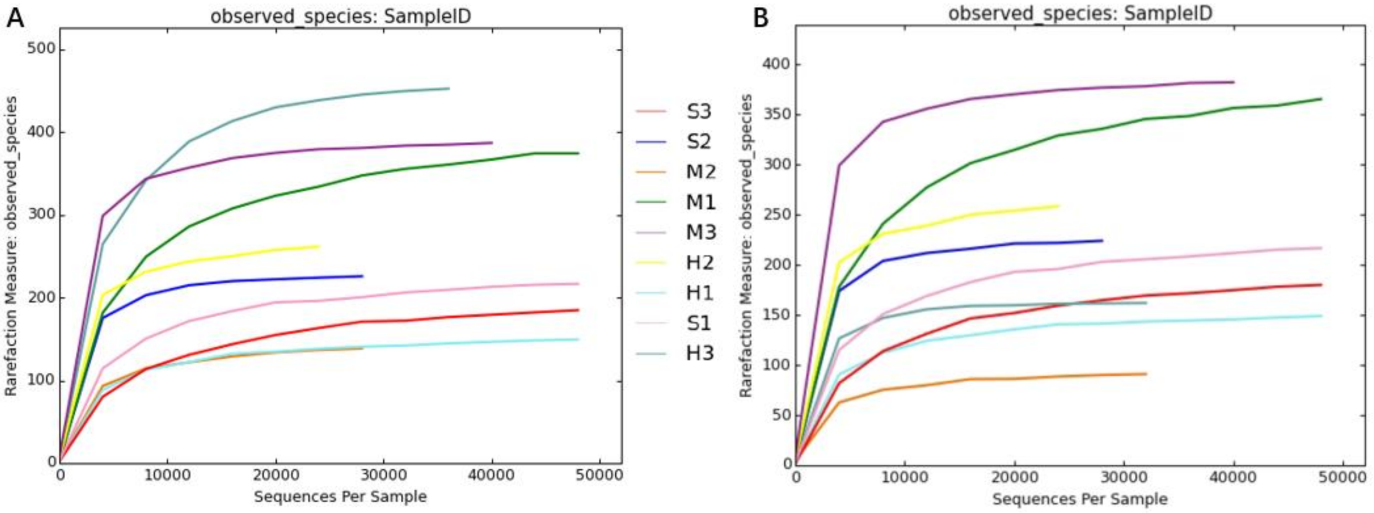


**Figure S1. Rarefaction curve of bacterial (A) and fungal (B) species under the three host conditions.** H: healthy aphids, M: mycotized aphids; S: starved aphids. Three replicates were set up for each condition.

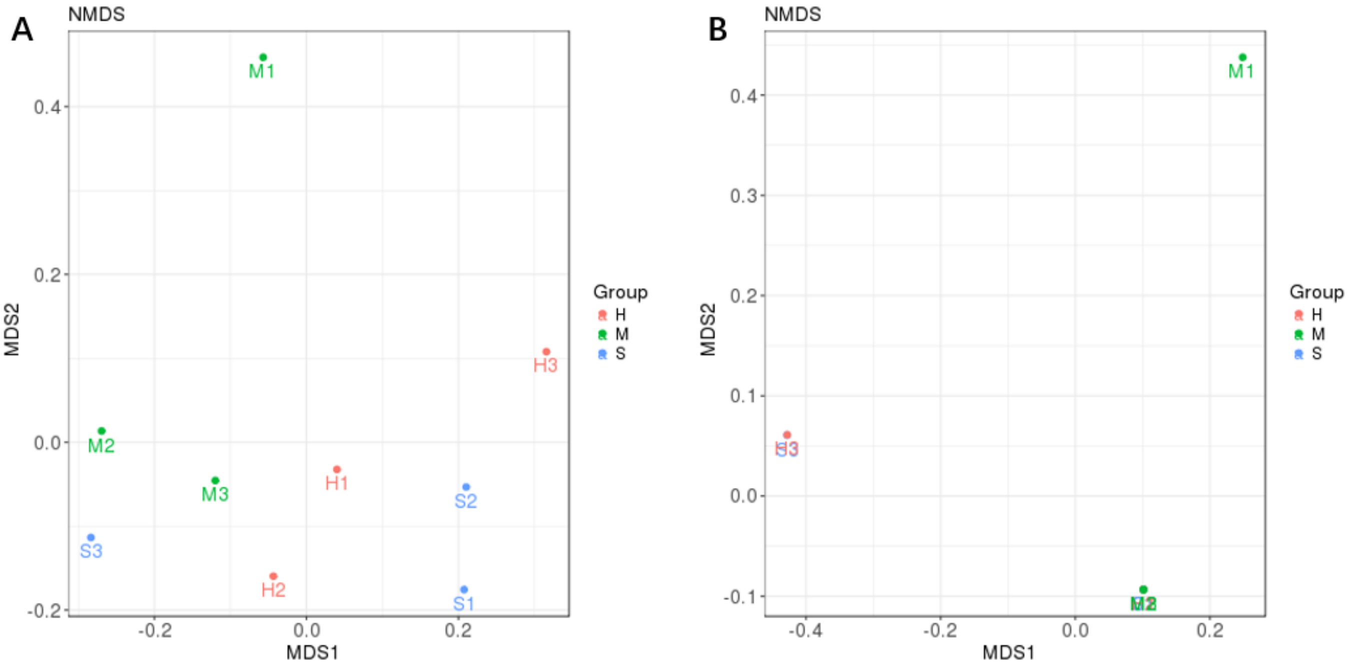


**Figure S2. NMDS analysis of aphid-associated bacterial (A) and fungal (B) flora under the three different conditions.** H: healthy aphids, M: mycotized aphids; S: starved aphids. The points in the figure represent the samples, and the distance between the points indicates the degree of difference.

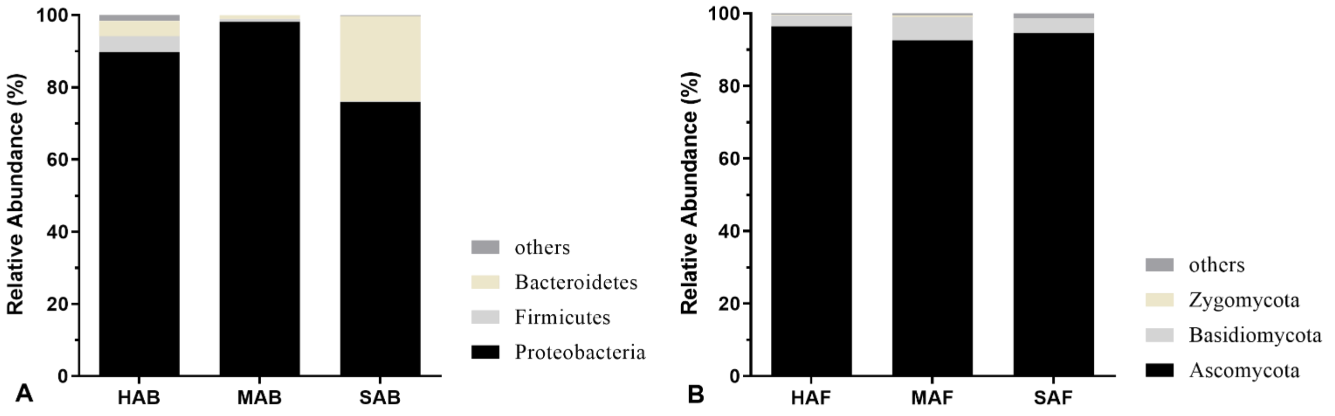


**Figure S3. Relative composition of bacterial (A) and fungal (B) phyla in aphid-associated microbial communities under the three different conditions.** HAB and HAF indicate healthy aphids, MAB and MAF mycotized aphids, SAB and SAF starved aphids.

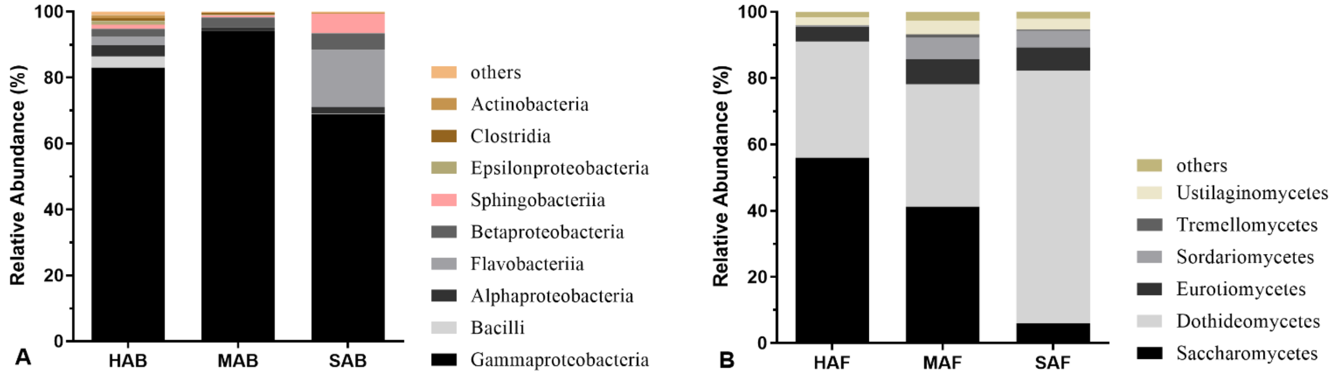


**Figure S4. Relative composition of bacterial (A) and fungal (B) classes in aphid-related microbiomes under the three different conditions.** HAB and HAF indicate healthy aphids, MAB and MAF mycotized aphids, SAB and SAF starved aphids.

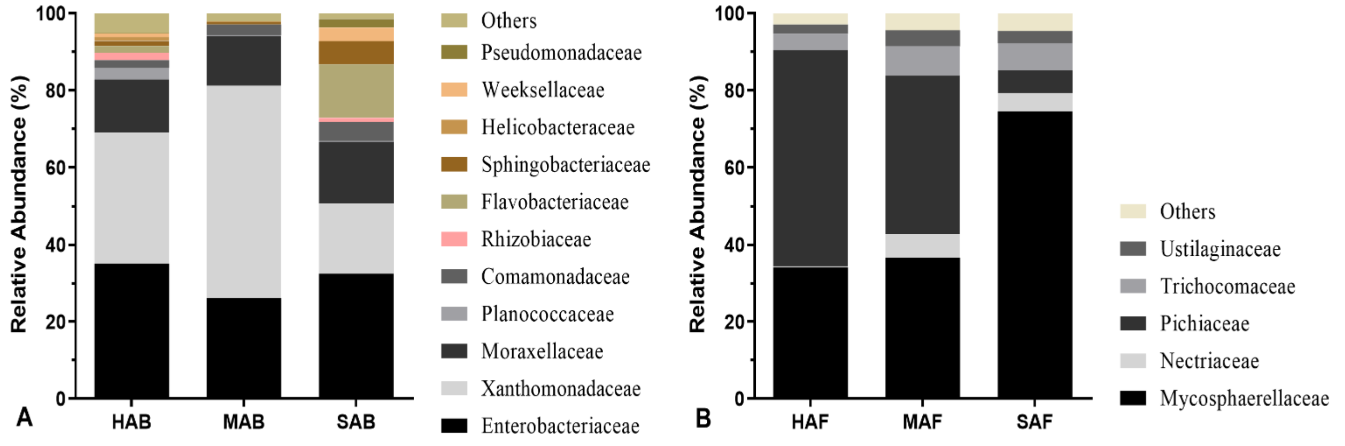


**Figure S5. Relative compositions of bacterial (A) and fungal (B) families in aphid-related microbiomes under the three different conditions.** HAB and HAF indicate healthy aphids, MAB and MAF mycotized aphids, SAB and SAF starved aphids.
